# Amino acid vectorization of salicylic acid enables efficient activation of NPR1‐dependent defense without phytotoxicity

**DOI:** 10.1002/ps.70800

**Published:** 2026-04-06

**Authors:** Ruth Oussou, Benoit Guichard, Loïc Lemée, Vincent Lebeurre, Cécile Marivingt‐Mounir, Jean‐François Chollet, Sylvain La Camera

**Affiliations:** ^1^ Institut de Chimie des Milieux et des Matériaux de Poitiers (IC2MP), Unité Mixte de Recherche CNRS 7285 Université de Poitiers Poitiers France; ^2^ Laboratoire Écologie & Biologie des Interactions (EBI), Unité Mixte de Recherche CNRS 7267 Université de Poitiers Poitiers France

**Keywords:** *Arabidopsis thaliana*, bacterial speck disease, plant innate immunity, prodrug strategy, PR1 induction, structure–activity relationship

## Abstract

**BACKGROUND:**

Addressing global food security under rapid population growth and climate change requires sustainable strategies to protect crops from pathogens while reducing reliance on agrochemicals. Salicylic acid (SA), a key phytohormone in plant defense, is a promising natural elicitor for crop protection. However, its rapid metabolism and phytotoxicity restrict practical use. To overcome these limitations, we developed SA–amino acid conjugates designed to improve protective efficacy of SA while minimizing its toxicity.

**RESULTS:**

Six SA–amino acid conjugates were synthesized through optimized multistep routes using either diaminocarboxylic acids or l‐glutamic acid with various linkers. Their defense‐inducing activity was evaluated in *Arabidopsis thaliana* (L.) Heynh. (*PR1::GUS* reporter line) and in disease‐resistance assays against *Pseudomonas syringae* pv. *tomato* (Okabe) Young *et al.* and *Pseudomonas syringae* pv. *maculicola* (McCulloch) Young *et al*. Conjugates **3a** and **7a** efficiently activated *PR1* expression and conferred NPR1‐dependent resistance comparable to free SA, while preventing SA‐associated phytotoxicity. The other conjugates showed lower or no activity, revealing a clear structure–activity relationship.

**CONCLUSION:**

This study highlights SA–amino acid conjugates as effective, non‐phytotoxic inducers of plant immunity. Structure–activity analyses indicate that both the nature of the SA–amino acid linkage and the properties of the amino acid side chain modulate biological activity, induction kinetics, and toxicity. Conjugates **3a** and **7a** combine strong protective efficacy with the absence of SA‐associated phytotoxicity, supporting amino acid vectorization as an effective approach for safer and controlled SA delivery and the rational design of next‐generation defense stimulators for sustainable crop protection. © 2026 The Author(s). *Pest Management Science* published by John Wiley & Sons Ltd on behalf of Society of Chemical Industry.

## Graphical abstract

Amino acid‐vectorized salicylic acid activates PR1/NPR1‐dependent defenses without phytotoxicity, showing an efficient strategy for controlled delivery and enhanced plant immunity.

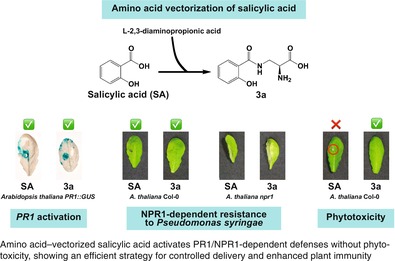

## 1 INTRODUCTION

In the context of accelerating population growth, current projections estimate that the global population will approach ten billion within the next three decades, with nutritional demands expected to rise accordingly.^1^, ^2^ Combined with climate change, this situation presents modern agriculture with a critical challenge: increasing productivity while minimizing environmental and health impacts.^3^ Achieving this goal requires effective control of plant pathogens that causes major yield losses. For decades, crop protection has relied largely on synthetic pesticides, which expanded rapidly after World War II with advances in organic chemistry.^4^ However, their adverse effects on the environment and human health have become increasingly evident, leading to public concern and motivating the search for safer and more sustainable alternatives.^5^, ^6^, ^7^

Salicylic acid (SA) is a key phytohormone involved in diverse biological processes, including plant development, flowering, and responses to both biotic and abiotic stresses.^8^, ^9^ Its functions are particularly associated with defense against biotrophic and hemibiotrophic pathogens, as its biosynthesis is activated downstream of pathogen recognition.^10^, ^11^ Accumulation of SA in the cytoplasm is indispensable for the activation of defense genes and the production of defense‐related metabolites.^12^ In particular, SA induces the expression of pathogenesis‐related (PR) proteins and systemic acquired resistance (SAR).^13^

The pivotal role of SA in plant immunity has been demonstrated by exogenous applications.^14^, ^15^ For instance, treatments of *NahG* transgenic plants – expressing a bacterial salicylate hydroxylase gene that prevents endogenous SA accumulation – restores SAR upon exogenous SA or analog treatment.^16^ SA accumulation is therefore essential for the induction of PR proteins, particularly PR‐1, and for NPR1‐mediated activation of SA‐responsive genes.^11^, ^17^ NPR1, normally present as a cytoplasmic oligomer, undergoes redox‐dependent monomerization in response to elevated SA levels, enabling its translocation to the nucleus where it interacts with TGA transcription factors to activate defense gene expression.^11^, ^18^ Mutant plants deficient in SA biosynthesis further confirm the necessity of SA accumulation for activating defense and establishing SAR.^13^ Because it is a naturally occurring compound with limited environmental persistence, SA represents an attractive lead for developing eco‐friendly crop protection agents.^19^, ^20^ However, exogenously applied SA is rapidly metabolized and compartmentalized in plant tissues,^21^ and can be phytotoxic in both monocots^22^, ^23^ and dicots.^24^, ^25^ These drawbacks have prompted research on SA analogs exhibiting lower phytotoxicity, greater stability, and improved resistance‐inducing capacity.^20^, ^26^, ^27^

Recently, we developed a vectorization strategy for SA based on the prodrug concept, in which SA is covalently coupled to nutrients such as amino acids or glucose that act as vectors recognized by endogenous plant transport systems. This approach facilitates SA translocation to target tissues and enables its gradual release, thereby improving both stability and bioavailability.^28^ In maize (*Zea mays* L.), a SA–l‐glutamic acid conjugate linked via a 1,2,3‐triazole ring exhibited phloem mobility in the *Ricinus* model comparable to free SA despite greater steric hindrance, and conferred local and systemic protection in maize plants infected with *Fusarium graminearum* or *Bipolaris maydis*. In response to *B. maydis*, this conjugate also up‐regulated two defense‐related genes, *ZmNPR1* and *ZmPR1*, confirming its ability to trigger systemic defense responses.^28^

The use of nutrient‐based vectors thus offers a promising route to enhance SA bioavailability and efficacy. In our previous work, we described a four‐step synthesis of SA or chlorinated analog conjugates with l‐glutamic acid or *β*‐d‐glucose via a 1,2,3‐triazole linker. Although all these conjugates are potential transporter substrates, their mobility and protective activity differed markedly: the SA–l‐glutamic acid conjugate showed superior phloem transport and greater protection than its glucose analog. Similarly, Wu *et al*. reported that a fenpiclonil–glutamic acid conjugate displayed improved phloem translocation compared with the glucose‐based counterpart^29^ and that the structure of the spacer influenced mobility.^30^ Overall, amino acid conjugation appears to provide more favorable transport properties than sugar conjugation.

In the present study, we report on an optimized multi‐step synthesis of novel SA–amino acid conjugates designed to improve defense activation while reducing the phytotoxic effects associated with exogenous SA application. Diaminocarboxylic acids were selected as coupling partners because their additional amino group allows covalent linkage to SA while preserving the free α‐amino acid function necessary for transporter recognition.^31^ The same design rationale guided the use of l‐glutamic acid, whose side‐chain carboxyl group enables conjugation while maintaining a free α‐amino acid group. To explore structure–activity relationships, three diaminocarboxylic acids of varying chain length were used without a spacer, while three l‐glutamic acid conjugates were synthesized: two in which SA or its mono‐chlorinated analog is linked to l‐glutamic acid via an ethylene glycol spacer, and a third in which SA is connected to l‐glutamic acid through a 1,2,3‐triazole‐containing linker. Defense activation was evaluated through *PR1* expression in transgenic *Arabidopsis thaliana* (L.) Heynh PR1::GUS reporter lines, and protective effects were assessed in *A. thaliana* Col0 and *npr1* mutant challenged with *Pseudomonas syringae* pv. *tomato* (Okabe) Young *et al*. strain DC3000 (Pst) and *Pseudomonas syringae* pv. *maculicola* (McCullough) Young *et al*. (Psm), both causal agents of bacterial speck disease.^17^, ^18^, ^32^ In addition, the six conjugates were evaluated for their *in vitro* antimicrobial activity against *P. syringae*.

## 2 MATERIALS AND METHODS

### 2.1 Chemical materials

All solvents used for organic synthesis were purchased from Acros Organics (Fisher Scientific SAS, Illkirch, France). Reagents were purchased from the following suppliers: Acros Organics (potassium carbonate, sodium hydroxide (NaOH)), TCI Europe N.V. (Paris, France; 2‐bromoethanol, 1‐(3‐dimethylaminopropyl)‐3‐ethylcarbodiimide hydrochloride (EDCl), *N‐α*‐Boc‐diaminocarboxylic acid, *N*‐Boc‐l‐glutamic acid 1‐*tert*‐butyl ester), Alfa Aesar (Thermo Fisher GmbH, Kandel, Germany; 4‐dimethylaminopyridine (DMAP), acetic anhydride, sodium acetate), Sigma‐Aldrich (Merck KGaA, Darmstadt, Germany; SA, aspirin, 5‐chlorosalicylic acid, 2‐(*N*‐morpholino)ethanesulfonic acid monohydrate (MES buffer)), Solarbio (Solarbio Science & Technology Co., Ltd, Beijing, China; surfactant Tween‐80).

### 2.2 Synthesis

Some reactions were performed under an inert nitrogen atmosphere to prevent interference from moisture or oxygen. Reaction progress was monitored by thin‐layer chromatography (TLC) using Merck silica gel 60F_254_ plates (Merck, Darmstadt, Germany). Purification was carried out by column chromatography on Merck silica gel 60 (particle size 0.015–0.04 mm). Melting points were determined using a Büchi B‐540 apparatus (Büchi Labortechnik AG, Flawil, Switzerland) and are reported uncorrected. Optical rotations were measured at 20 °C using an Anton Paar MCP100 polarimeter (software version 1.10.3399.115; Anton Paar GmbH, Graz, Austria) equipped with a light‐emitting diode (LED) light source (*λ* = 589 nm). Samples were dissolved in 1 mL of 6 m hydrochloric acid (HCl) and diluted to 10 mL with ultrapure water (final HCl concentration 0.6 m). Concentrations (*c*) are expressed in g 100 mL^−1^ and correspond to the exact weighed amounts of compound. Reported values are specific rotations ${\left[\alpha \right]}_{D}^{20}$. Due to their limited solubility in aqueous acidic medium, reliable optical rotation values could not be obtained for compounds **7a** and **7b**. Proton (^1^H) and carbon‐13 (^13^C) nuclear magnetic resonance (NMR) spectra were recorded in deuterated chloroform (CDCl_3_) or deuterated dimethyl sulfoxide (DMSO‐*d*_6_) solvents on Bruker AVANCE 400 MHz or Bruker Ultrashield™ 500 Avance NEO spectrometers (Bruker, Karlsruhe, Germany), operating at 400 or 500 MHz for proton and 101 or 126 MHz for carbon nuclei, respectively. DEPT (distortionless enhancement by polarization transfer, 90 and 135) and two‐dimensional (2D) NMR experiments (^1^H–^13^C heteronuclear single quantum correlation/heteronuclear multiple‐bond correlation (HSQC/HMBC) and ^1^H–^1^H correlation spectroscopy (COSY)) were used to assist signal assignment. Chemical shifts (δ) are reported in parts per million (ppm) relative to tetramethylsilane (TMS) as internal standard, and coupling constants (*J*) are given in hertz (Hz). Multiplicities are designated as s (singlet), bs (broad singlet), d (doublet), t (triplet), dd (doublet of doublets), td (triplet of doublets), and m (multiplet). High‐resolution mass spectroscopy (HRMS) spectra were obtained using a Bruker Q‐TOF Impact HD mass spectrometer equipped with an electrospray ionization (ESI) source.

### 2.3 Biological materials and growth conditions

Experiments were conducted using *A. thaliana* Col‐0 (wild‐type), the *npr1* (*nonexpressor of PR1 gene*) mutant,^32^ and the *PR1::GUS* reporter line (ABRC stock number: CS6357).^32^ This reporter line carries a transcriptional construct that accurately reflects expression of pathogenesis‐related gene one (*PR1*) through the beta‐glucuronidase (*GUS*) activity. Plants were grown in an autoclaved mix of compost/vermiculite (3:1) and placed in a phytotron under controlled conditions (22 °C, 65% relative humidity, 9 h photoperiod) for 6–7 weeks. The plants were watered twice a week by adding approximately 1 L of water to each tray containing 15 plant pots.

Two bacterial pathogens were used: Pst and Psm. Bacteria were streaked from −80 °C glycerol stocks onto low‐salt Luria–Bertani (LB) agar plates (10 g L^−1^ tryptone, 5 g L^−1^ yeast extract, 5 g L^−1^ sodium chloride (NaCl), pH 7.0) supplemented with rifampicin (50 μg mL^−1^) and incubated at 28 °C for 2 days. Fresh colonies were then inoculated into liquid LB medium supplemented with rifampicin (50 μg mL^−1^) and cultured at 28 °C with shaking for 24 h until mid‐ to late‐log phase (optical density measured at 600 nm (OD_600_) = 0.6–1.0).

### 2.4 Histochemical GUS assays

The GUS staining procedure was adapted from published protocols.^17^, ^32^ Each compound, including SA, was first dissolved successively in 1 mL of 6 m HCl and 1 mL of absolute ethanol, then diluted to a final volume of 10 mL with MES buffer (10 mm, pH 5.0, containing 0.05% (*v/v*) Tween‐80), yielding 10 mm stock solutions. Working solutions (1 mm) were prepared by dilution in the same MES buffer. Controls consisted of the HCl/ethanol/MES mixture prepared without compound. Six‐week‐old *PR1::GUS* plants were treated by applying 10 μL of each 1 mm solution directly onto the leaf surface. Leaves were harvested at 24, 48, and 72 h post‐treatment and placed in six‐well plates. Subsequently, 2 mL of GUS staining solution (1 mm X‐Gluc prepared at 25 mg mL^−1^ in dimethylformamide; 100 mm NaPi buffer, pH 7.0; 0.5 mm potassium ferricyanide/ferrocyanide; 10 mm ethylenediaminetetraacetic acid (EDTA); 0.1% (*v/v*) Triton X‐100) were added per well. To facilitate infiltration, plates were placed under partial vacuum (−300 mbar below atmospheric pressure) for 30 min in a glass desiccator connected to a vacuum pump. Plates were then sealed and incubated at 37 °C for 24 h. After incubation, tissues were de‐stained by successive washes with 95% and 70% ethanol for several hours. Blue staining was visualized and imaged using an Amersham™ Imager 600 (Cytiva, Marlborough, MA, USA). Three independent biological experiments were performed. In each experiment, three individual plants were used per treatment and per time. At 24, 48, and 72 h post‐treatment, three leaves were collected from each of three distinct plants (i.e., nine leaves per treatment and per time point). For each time point, a new set of plants was used to avoid stress effects caused by previous leaf sampling.

#### 2.4.1 Semi‐quantitative analysis of GUS staining

GUS‐stained leaves were imaged under identical lighting and magnification conditions. For semi‐quantitative analysis, six leaves per treatment and time point (two leaves from each of three independent plants) were analyzed from one representative experiment. Images were processed using ImageJ2 software version 2.16.0/1.54p. The blue‐stained area was quantified by applying identical threshold settings to all images, and the percentage of thresholded blue‐stained area was calculated relative to a selected region of interest encompassing the leaf.

### 2.5 Phytotoxicity and disease assessment

Six‐week‐old *A. thaliana* Col‐0 plants were treated with 10 μL of each 1 mm compound or control solution applied to the leaf surface. Phytotoxicity symptoms were evaluated 3 days post‐treatment. Leaves were then collected and photographed using a Canon EOS 700D camera (Canon, Tokyo, Japan). For each treatment, nine leaves (three leaves collected from each of three individual plants) were analyzed per experiment. The experiment was repeated independently three times.

For disease assessment, six‐week‐old *A. thaliana* Col‐0 and *npr1* plants were treated similarly. Two days later, half of each treated leaf was infiltrated with a bacterial suspension (OD_600_ = 0.2; ~10^8^ CFU mL^−1^) prepared in 10 mm magnesium chloride (MgCl_2_) using needleless syringe.^17^, ^33^ Disease symptoms were evaluated 2 days after infection, and leaves photographed as earlier. Each replicate consisted of nine leaves from three plants and experiments were performed using three independent replicates.

To provide a quantitative assessment of disease symptoms observed, a disease severity index (DSI) was established based on visual scoring of leaf symptoms. Each infiltrated leaf was independently evaluated by four blinded observers using a 0–4 scale, where: 0 = no visible symptoms (green, turgid tissue, appearance comparable to uninfected controls), 1 = very mild diffuse chlorosis without necrosis, 2 = moderate chlorosis in the infected area with beginning loss of turgor but largely intact tissue, 3 = severe chlorosis with tissue softening and initial localized necrosis, and 4 = extensive necrosis and tissue collapse comparable to infected mock controls.

For each treatment, three biological replicates (three individual leaves) were scored. The final DSI value reported for each leaf corresponds to the mean of the four independent observers. Inter‐observer agreement was assessed using Kendall's coefficient of concordance (*W*).

Statistical analyses were performed using non‐parametric tests. Overall treatment effects were evaluated by Kruskal–Wallis tests, followed by Dunn's multiple comparison *post hoc* test with Benjamini–Hochberg false discovery rate (FDR‐BH) correction (*α* = 0.05). To compare the relative effectiveness of treatments against Pst and Psm, the concordance between median DSI values for both pathogens was assessed using Spearman's rank correlation.

### 2.6 *In vitro* antimicrobial tests

The antimicrobial activity of SA and SA conjugates against Pst and Psm was evaluated by monitoring bacterial growth in a microspectrophotometric assay.^34^ Solutions of each compound at 10 mm and the control solution were sterilized by filtration through 0.22 μm membranes. In sterile 96‐well plates, 20 μL of each 10 mm compound solution, control solution, or 70% ethanol was added to 180 μL of fresh LB culture (initial OD_600_ = 0.01), giving a final compound concentration of 1 mm. Plates were sealed and incubated in a Synergy H1 BioTek microplate reader (Bio‐Tek, Winooski, VT, USA) at 28 °C with continuous orbital shaking. Absorbance was recorded at 600 nm every 30 min for 24 h. Each condition was tested with 6 to 12 technical replicates, and four biological replicates. Data processing included baseline subtraction, averaging technical replicates, and representing bacterial growth as log_10_(absorbance at 600 nm) over 24 h.

## 3 RESULTS AND DISCUSSION

### 3.1 Synthesis of salicylic acid–amino acid conjugates

**Figure 1:**
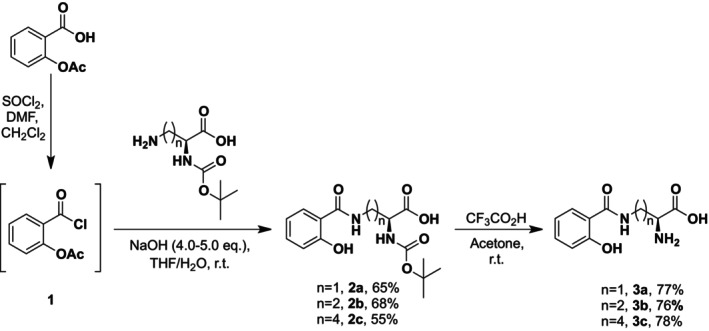
Synthetic pathway for the preparation of SA‐based conjugates **3a–3c** with diaminocarboxylic acids.

**Figure 2:**
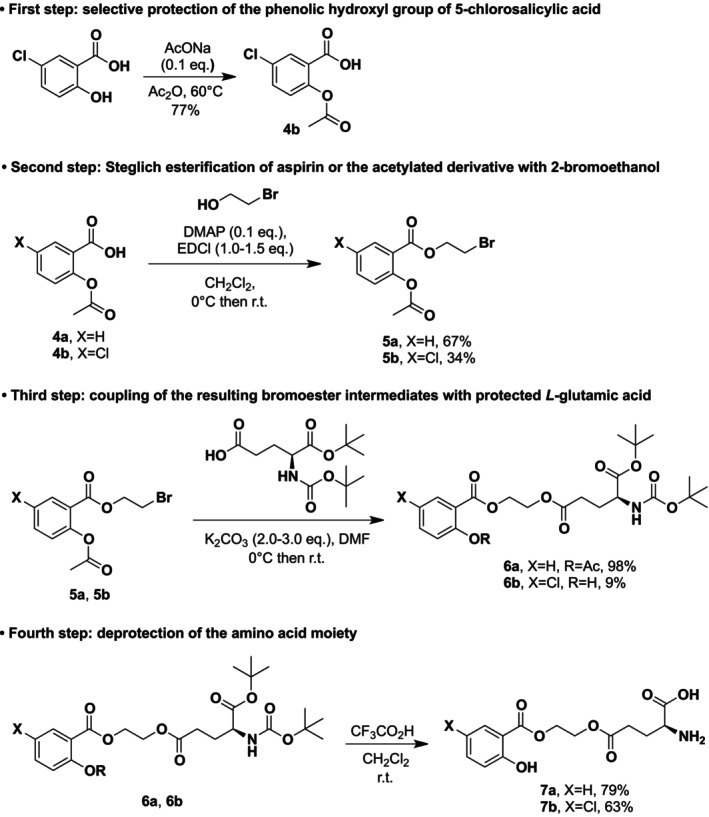
Synthetic pathway for the preparation of SA and 5‐chlorosalicylic acid–l‐glutamic acid conjugates **7a** and **7b** using an ethylene glycol spacer. AcONa: sodium acetate; Ac_2_O: acetic anhydride; DMAP: 4‐dimethylaminopyridine; EDCl: 1‐ethyl‐3‐(3‐dimethylaminopropyl)carbodiimide hydrochloride; DMF: *N,N*‐dimethylformamide.

**Figure 3:**
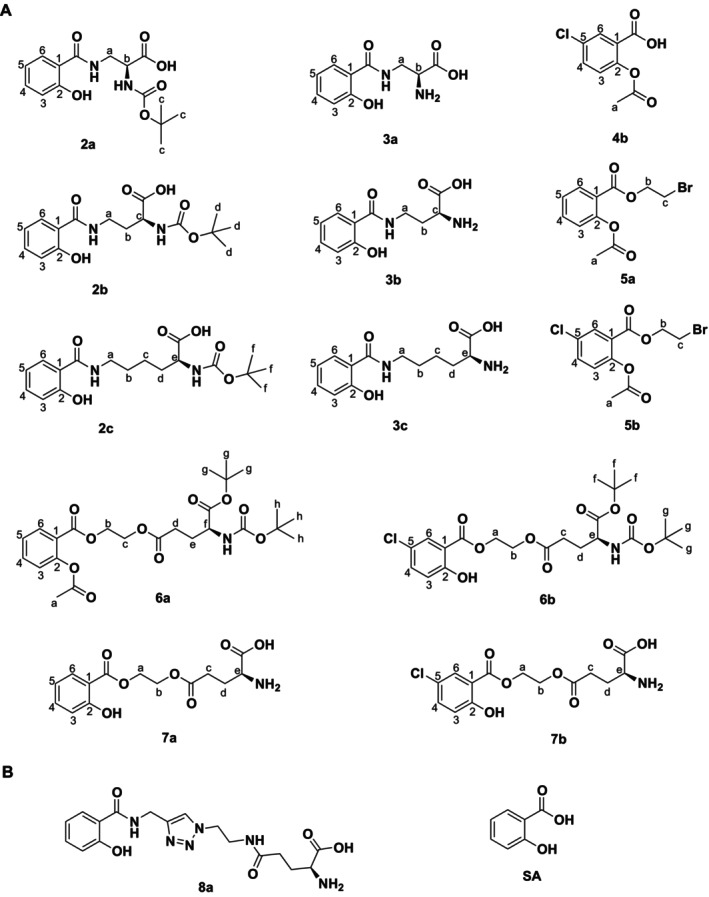
Atom numbering scheme of SA derivatives used for ^1^H and ^13^C NMR assignments (A), and chemical structures of compound **8a** and SA (B).

This study focuses on a series of six SA–amino acid conjugates comprising: (i) three SA conjugates with diaminocarboxylic acids lacking a spacer arm (Fig. 1, compounds **3a**–**3c**), (ii) two conjugates in which SA or a mono‐halogenated analog is linked to l‐glutamic acid through an ethylene glycol spacer (Fig. 2, compounds **7a** and **7b**), and (iii) the SA–l‐glutamic acid conjugate incorporating a 1,2,3‐triazole spacer (Fig. 3(B), compound **8a**), synthesized as previously reported and re‐characterized in the present study; ${\left[\alpha \right]}_{D}^{20}$ = +22.1 (*c* = 0.104, 0.6 m HCl).^28^

The three SA conjugates lacking a spacer arm were synthesized through the following sequence: (i) preparation of the acid chloride derivative of aspirin; (ii) peptide coupling between this acid chloride and Boc‐protected diaminocarboxylic acids under basic conditions, affording coupled intermediates while simultaneously removing the acetyl group from the phenol function; (iii) final acidic deprotection, yielding the target conjugates bearing a free α‐amino acid moiety.

The synthesis of SA (or its mono‐chlorinated analog)–l‐glutamic conjugates incorporating an ethylene glycol spacer proceeded as follows: (i) selective protection of the phenolic hydroxyl group of 5‐chlorosalicylic acid (ii) Steglich esterification of aspirin or acetylated 5‐ClSA with 2‐bromoethanol to generate bromoester intermediates; (iii) nucleophilic substitution of these intermediates with the free carboxyl group of protected l‐glutamic acid, giving the protected conjugates; (iv) acidic deprotection of the carboxyl, amino, and phenolic functions, affording fully deprotected conjugates with a free α‐amino acid group.

This series of six conjugates provides a coherent framework for subsequent structure–activity relationship analysis. The bioactive component is SA or its mono‐halogenated analog, while the vectorization moiety is an *α‐*amino acid recognizable by plant transport systems. Regarding spacer diversity, ethylene glycol is attached to the active moiety via an ester bond, whereas the 1,2,3‐triazole ring is linked through an amide bond.

#### 3.1.1 Synthesis of the three SA–diaminocarboxylic acids conjugates (Figs 1 and 3(A); compounds **3a**–**3c**)

The three conjugates combine SA with l‐2,3‐diaminopropionic acid (*n* = 1, **3a**), l‐2,4‐diaminobutyric acid (*n* = 2, **3b**), or l‐lysine (*n* = 4, **3c**). In all cases, these conjugates (**3a–3c**) are functionalized through an amide bond formed between the carboxyl group of SA and the side‐chain amino group of the corresponding amino acid. The carboxylic acid group of aspirin was first converted into the corresponding acid chloride (**1**) following adapted procedures from the literature.^35^ Thus, to a solution of aspirin (0.54 g, 4 mmol) in anhydrous dichloromethane (CH_2_Cl_2_, 10 mL), thionyl chloride (SOCl_2_, 1 mL, 13.78 mmol) was added. The mixture was refluxed under nitrogen for 2 h. After evaporation under reduced pressure, the resulting crude acetylsalicyloyl chloride (**1**) was immediately dissolved in anhydrous tetrahydrofuran (THF) and reacted with the appropriate *N‐α*‐Boc‐protected diaminocarboxylic acid to give intermediates **2a–2c**.

##### 3.1.1.1 General procedure for the synthesis of intermediates **2a–2c**

*N‐α*‐Boc‐diaminocarboxylic acid (1.0 equiv.) was dissolved in an aqueous NaOH solution (4.0–5.0 equiv.). The solution of acid chloride **1** was added dropwise to the aqueous phase under continuous stirring at room temperature, and the reaction mixture was stirred until complete conversion of both reactants. The mixture was then acidified to pH 3 with a solution of HCl 1 mm and extracted with ethyl acetate (EtOAc, 3 × 30 mL). The combined organic phases were dried over sodium sulfate (Na_2_SO_4_), filtered, and concentrated under reduced pressure. The crude material was purified by silica gel column chromatography (CH_2_Cl_2_/EtOAc 4:1) to afford intermediates **2a–2c**.

###### 3.1.1.1.1 2*S*‐[(*tert*‐Butoxycarbonyl)amino]‐3‐(2‐hydroxybenzamido)propanoic acid (**2a**)

Compound **2a** (1.14 g, 3.50 mmol, 65%) was obtained by reacting acid chloride **1** (1.0 equiv.) with 2*S*‐(*tert*‐butoxycarbonylamino)‐3‐aminopropionic acid (1.10 g, 5.39 mmol, 1.0 equiv.). It was isolated as a yellow oil.

Retention factor (Rf) = 0.24 (EtOAc). ^1^H NMR (400 MHz, DMSO‐*d*_6_): δ 14.10 (bs, 1H, OH), 12.47 (s, 1H, OH), 8.65 (t, ^3^ *J* = 5.3 Hz, 1H, NH), 7.87 (d, ^3^ *J* = 7.9 Hz, 1H, NH), 7.45 (td, ^3^ *J* = 8.2 Hz, 1H, H_4_), 7.16 (d, ^3^ *J* = 8.3 Hz, 1H, H_6_), 6.97 (m, 2H, H_3_ and H_5_), 3.89 (m, 1H, H_b_), 3.46 (dd, ^2^ *J* = 12.0 Hz, ^3^ *J* = 5.3 Hz, 2H, H_a_), 1.38 (s, 9H, H_c_). ^13^C NMR (101 MHz, DMSO‐*d*_6_): δ 172.2, 169.5 (2CO), 160.4 (C, C_2_), 156.3 (CO_carbamate_), 133.8 (CH, C_4_), 128.4 (CH, C_6_), 118.6 (CH, C_5_), 117.4 (CH, C_3_), 115.3 (C, C_1_), 78.8 (_C*tert*‐butyl_), 52.4 (CH, C_b_), 37.4 (CH_2_, C_a_), 28.9 (3 CH_3_, C_c_).

###### 3.1.1.1.2 2*S*‐[(*tert*‐Butoxycarbonyl)amino]‐4‐(2‐hydroxybenzamido)butanoic acid (**2b**)

Compound **2b** (0.52 g, 3.50 mmol, 68%) was isolated as an orange oil. It was obtained by reacting acid chloride **1** (1.0 equiv.) with 2‐(*tert*‐butoxycarbonylamino)‐4‐aminobutyric acid (0.50 g, 2.27 mmol, 1.0 equiv.).

Rf = 0.32 (EtOAc). ^1^H NMR (400 MHz, DMSO‐*d*_6_): δ 12.59 (s, 2H, 2 OH), 8.80 (t, ^3^ *J* = 5.0 Hz, 1H, NH), 7.84 (d, ^3^ *J* = 7.6 Hz, 1H, NH), 7.50–7.30 (td, ^3^ *J* = 8.1 Hz, 1H, H_4_), 7.16 (d, ^3^ *J* = 8.1 Hz, 1H, H_6_), 6.88 (m, 2H, H_3_ and H_5_), 3.98 (m, 1H, H_c_), 3.36 (dd, ^2^ *J* = 12.4 Hz, ^3^ *J* = 6.4 Hz, 2H, H_a_), 2.02 (m, 1H, H_b_), 1.80 (m, 1H, H_b_), 1.38 (s, 9H, H_d_). ^13^C NMR (101 MHz, DMSO‐*d*_6_): δ 174.3, 169.5 (2CO), 160.6 (C, C_2_), 156.0 (CO_carbamate_), 134.1 (CH, C_4_), 128.1 (CH, C_6_), 118.9 (CH, C_5_), 117.8 (CH, C_3_), 115.6 (C, C_1_), 78.5 (C_*tert*‐butyl_), 51.8 (CH, C_c_), 36.6 (CH_2_, C_a_), 30.8 (CH_2_, C_b_), 28.7 (3 CH_3_, C_d_).

###### 3.1.1.1.3 2*S*‐[(*tert*‐Butoxycarbonyl)amino]‐6‐(2‐hydroxybenzamido)hexanoic acid (**2c**)

Compound **2c** (1.82 g, 4.58 mmol, 55%) was isolated as an orange oil. The product was obtained by reacting acid chloride **1** (1.0 equiv.) with 2*S*‐(*tert*‐butoxycarbonylamino)‐6‐aminohexanoic acid (2.23 g, 9.05 mmol, 1.0 equiv.).

Rf = 0.29 (EtOAc). ^1^H NMR (400 MHz, DMSO‐*d*_6_): δ 12.74 (s, 1H, OH), 12.43 (bs, 1H, OH), 8.82 (t, ^3^ *J* = 5.5 Hz, 1H, NH), 7.86–7.81 (dd, ^3^ *J* = 8.4 Hz, ^4^ *J* = 1.4 Hz, 1H, H_6_), 7.40 (td, ^3^ *J* = 8.0 Hz, ^4^ *J* = 1.6 Hz, 1H, H_4_), 7.06 (d, ^3^ *J* = 8.0 Hz, 1H, NH), 6.94–6.84 (m, 2H, H_3_ and H_5_), 3.85 (m, 1H, He), 3.28 (m, 2H, H_a_), 1.79–1.50 (m, 4H, H_b_ and H_d_), 1.37 (m, 11H, H_c_ and H_f_). ^13^C NMR (101 MHz, DMSO‐*d*_6_): δ 174.7, 169.5 (2CO), 160.8 (C, C_2_), 156.1 (CO_carbamate_), 134.1 (CH, C_4_), 127.9 (CH, C_6_), 118.1 (CH, C_5_), 117.9 (CH, C_3_), 115.5 (C, C_1_), 78.4 (C_*tert*‐butyl_), 53.8 (CH, C_e_), 39.2 (CH_2_, C_a_), 30.9, 28.9 (2CH_2_, C_b_ and C_d_), 28.7 (3 CH_3_, C_f_), 23.6 (CH_2_, C_c_).

##### 3.1.1.2 Deprotection of the α‐amino acid function of intermediates **2a–2c** to yield conjugates **3a–3c**

Deprotection of intermediates **2a–2c** under acidic conditions removed the *tert*‐butoxycarbonyl protecting groups from the α‐amino acid functions, giving the desired conjugates **3a–3c**. Trifluoroacetic acid (CF_3_CO_2_H, 5–7 mL) was added to a solution of intermediates **2a–2c** in acetone (10–20 mL). The reaction mixture was stirred at room temperature until complete consumption of the starting materials. Evaporation under reduced pressure afforded conjugates **3a–3c** in 77%, 76%, and 78% yield, respectively.

###### 3.1.1.2.1 2*S*‐Amino‐3‐(2‐hydroxybenzamido)propanoic acid (**3a**)

Compound **3a** (0.17 g, 0.77 mmol, 77%) was obtained as a beige solid by treating compound **2a** (0.34 g, 1.00 mmol, 1.0 equiv.) with trifluoroacetic acid (2 mL) in acetone (10 mL) under stirring at room temperature for 2 h.

Rf = 0.03 (EtOAc); melting point (mp) = 92–94 °C; ${\left[\alpha \right]}_{D}^{20}$ = −37.9 (*c* = 0.211, 0.6 m HCl). ^1^H NMR (400 MHz, DMSO‐*d*_6_): δ 9.21 (s, 1H, NH), 7.86 (d, ^3^ *J* = 7.7 Hz, 1H, H_6_), 7.39 (t, ^3^ *J* = 8.0 Hz, 1H, H_4_), 6.97 (d, ^3^ *J* = 8.0 Hz, 1H, H_3_), 6.88 (t, ^3^ *J* = 7.7 Hz, 1H, H_5_), 3.79 (m, 2H, H_a_ and H_b_), 3.69 (m, 1H, H_a_). ^13^C NMR (101 MHz, DMSO‐*d*_6_): δ 169.6, 169.4 (2CO), 160.0 (C, C_2_), 134.0 (CH, C_6_), 129.0 (CH, C_4_), 119.0 (CH, C_5_), 117.8 (CH, C_3_), 116.3 (C, C_1_), 53.5 (CH, C_b_), 40.3 (CH_2_, C_a_). HRMS (ESI, methanol): *m/z* calculated for C_10_H_13_N_2_O_4_ [M + H]^+^ 225.0870, *m/z* found 225.0873.

###### 3.1.1.2.2 2*S*‐Amino‐4‐(2‐hydroxybenzamido)butanoic acid (**3b**)

Compound **3b** (0.28 g, 1.17 mmol, 76%) was obtained as a beige solid by treating compound **2b** (0.52 g, 1.54 mmol, 1.0 equiv.) with trifluoroacetic acid (3 mL) in acetone (10 mL) under stirring at room temperature for 5 h.

Rf = 0.02 (EtOAc); mp = 108–110°C; ${\left[\alpha \right]}_{D}^{20}$ = +19.1 (*c* = 0.186, 0.6 m HCl). ^1^H NMR (500 MHz, DMSO‐*d*_6_): δ 14.10 (bs, 1H, OH), 12.41 (s, 1H, OH), 8.95 (t, ^3^ *J* = 5.4 Hz, 1H, NH), 8.34 (s, 2H, NH_2_), 7.82 (dd, ^3^ *J* = 8.0 Hz, ^4^ *J* = 1.4 Hz, 1H, H_6_), 7.40 (td, ^3^ *J* = 8.0 Hz, ^4^ *J* = 1.6 Hz, 1H, H_4_), 6.94–6.87 (m, 2H, H_3_ and H_5_), 4.08–3.88 (m, 1H, H_c_), 3.53–3.40 (m, 2H, H_a_), 2.11–2.01 (m, 2H, H_b_). ^13^C NMR (126 MHz, DMSO‐*d*_6_): δ 171.4, 169.5 (2CO), 160.65 (C, C_2_), 134.0 (CH, C_4_), 128.3 (CH, C_6_), 118.9, 117.8 (2CH, C_3_ and C_5_), 115.7 (C, C_1_), 52.7 (CH, C_c_), 37.1 (CH_2_, C_a_), 30.7 (CH_2_, C_b_). HRMS (ESI, methanol): *m/z* calculated for C_11_H_15_N_2_O_4_ [M + H]^+^ 239.1026, *m/z* found 239.1027.

###### 3.1.1.2.3 2*S*‐Amino‐6‐(2‐hydroxybenzamido)hexanoic acid (**3c**)

Compound **3c** (0.46 g, 1.69 mmol, 78%) was obtained as a beige solid by treating compound **2c** (0.81 g, 2.20 mmol, 1.0 equiv.) with trifluoroacetic acid (5 mL) in acetone (20 mL) under stirring at room temperature for 24 h.

Rf = 0.04 (EtOAc); mp = 145–147 °C; ${\left[\alpha \right]}_{D}^{20}$ = +8.7 (*c* = 0.357, 0.6 m HCl). ^1^H NMR (400 MHz, DMSO‐*d*_6_): δ 8.92 (t, ^3^ *J =* 5.3 Hz, 1H, NH), 7.87 (dd, ^3^ *J* = 7.9 Hz, ^4^ *J* = 1.2 Hz, 1H, H_6_), 7.38 (td, ^3^ *J* = 8.4 Hz, ^4^ *J* = 1.3 Hz, 1H, H_4_), 7.01–6.76 (m, 2H, H_3_ and H_5_), 3.59–3.40 (m, 1H, H_e_), 3.34–3.19 (m, 2H, H_a_), 1.89–1.65 (m, 2H, H_d_), 1.62–1.51 (m, 2H, H_b_), 1.49–1.29 (m, 2H, H_c_). ^13^C NMR (101 MHz, DMSO‐*d*_6_): δ 171.4, 169.3 (2CO), 160.6 (C, C_2_), 134.0 (CH, C_4_), 128.2 (CH, C_6_), 118.9, 117.8 0 (2CH, C_3_ and C_5_), 115.7 0 (C, C_1_), 53.8 0 (CH, C_e_), 39.2 (CH_2_, C_a_), 30.8 (CH_2_, C_d_), 29.0 (CH_2_, C_b_), 22.7 (CH_2_, C_c_). HRMS (ESI, methanol): *m/z* calculated for C_13_H_19_N_2_O_4_ [M + H]^+^ 237.1339, *m/z* found 237.1347.

#### 3.1.2 Synthesis of SA or mono‐halogenated SA conjugates with l‐glutamic acid using an ethylene glycol spacer (Figs 2 and 3(A); compounds **7a** and **7b**)

##### 3.1.2.1 Selective protection of the phenolic hydroxyl group of 5‐chlorosalicylic acid (**4b**)

Sodium acetate (0.1 equiv.) was added to a suspension of 5‐chlorosalicylic acid (4.00 g, 23.2 mmol, 1.0 equiv.) in acetic anhydride. The reaction mixture was heated at 60 °C for 3 h with stirring. After cooling, it was poured into chilled distilled water. The precipitate was collected by Büchner filtration, washed with cold distilled water, and dried in an oven at 60 °C to afford 2‐acetoxy‐5‐chlorobenzoic acid (**4b**) as a white powder (3.85 g, 17.9 mmol, 77% yield).

###### 3.1.2.1.1 2‐Acetoxy‐5‐chlorobenzoic acid (**4b**)

Rf = 0.18 (CH_2_Cl_2_/EtOAc 8:2). ^1^H NMR (400 MHz, DMSO‐*d*_6_): δ 13.44 (bs, 1H, OH), 7.89 (d,^4^ *J* = 2.7 Hz, 1H, H_6_), 7.71 (dd, ^3^ *J* = 8.6 Hz, ^4^ *J* = 2.7 Hz, 1H, H_4_), 7.26 (d, ^3^ *J* = 8.6 Hz, 1H, H_3_), 2.25 (s, 3H, H_a_). ^13^C NMR (101 MHz, DMSO‐*d*_6_): δ 169.0, 164.5 (2CO), 148.5 (C, C_2_), 133.5 (CH, C_4_), 130.7 (CH, C_6_), 130.1 (C, C_1_), 125.9 (C, C_5_), 125.9 (CH, C_3_), 20.7 (CH_3_, C_a_).

##### 3.1.2.2 Steglich esterification of acetylated derivatives with 2‐bromoethanol to yield intermediates **5a** and **5b**

A suspension of aspirin (**4a**) or compound **4b** (1.0 equiv.) in anhydrous CH_2_Cl_2_ cooled to 0 °C was treated with 2‐bromoethanol (1.0 equiv.), followed by sequential addition of DMAP (0.1 equiv.) and EDCl (1.0–1.5 equiv.). The reaction mixture was kept at 0 °C for 1 h, then allowed to warm to room temperature and stirred until complete conversion of compound **4a** or compound **4b** was achieved. The mixture was washed with cold distilled water (3 × 50 mL), dried over MgSO_4_, filtered, and concentrated under reduced pressure. Purification by silica gel column chromatography (pentane/CH_2_Cl_2_ 7:3, then CH_2_Cl_2_) afforded compounds **5a** and **5b**.

###### 3.1.2.2.1 2‐Bromoethyl 2‐acetoxybenzoate (**5a**)

Compound **5a** (3.63 g, 12.7 mmol, 67%) was obtained as a colorless oil after 60 h by reacting aspirin (3.40 g, 18.9 mmol, 1.0 equiv.) with 2‐bromoethanol (2.36 g, 18.9 mmol, 1.0 equiv.).

Rf = 0.90 (CH_2_Cl_2_). ^1^H NMR (400 MHz, CDCl_3_): δ 8.05 (dd, ^3^ *J* = 7.7 Hz, ^4^ *J* = 1.7 Hz, 1H, H_6_), 7.57 (td, ^3^ *J* = 8.1 Hz, ^4^ *J* = 1.7 Hz, 1H, H_4_), 7.33 (td, ^3^ *J* = 7.7 Hz, ^4^ *J* = 1.1 Hz, 1H, H_5_), 7.11 (dd, ^3^ *J* = 8.1 Hz, ^4^ *J* = 1.1 Hz, 1H, H_3_), 4.57 (t, ^3^ *J* = 6.1 Hz, 2H, H_b_), 3.70 (t, ^3^ *J* = 6.1 Hz, 2H, H_c_), 2.36 (s, 3H, H_a_). ^13^C NMR (101 MHz, CDCl_3_): δ 169.8, 164.0 (2CO), 151.0 (C, C_2_), 134.4 (CH, C_4_), 132.0 (CH, C_6_), 126.2 (CH, C_5_), 124.0 (CH, C_3_), 122.7 (C, C_1_), 64.5 (CH_2_, C_b_), 28.6 (CH_2_, C_c_), 21.2 (CH_3_, C_a_).

###### 3.1.2.2.2 2‐Bromoethyl 2‐acetoxy‐5‐chlorobenzoate (**5b**)

Compound **5b** (0.51 g, 1.82 mmol, 34%) was obtained as a colorless oil after 24 h by reacting compound **4b** (1.15 g, 5.36 mmol, 1.0 equiv.) with 2‐bromoethanol (0.67 g, 5.36 mmol, 1.0 equiv.).

Rf = 0.84 (CH_2_Cl_2_). ^1^H NMR (400 MHz, CDCl_3_): δ 8.02 (d, ^4^ *J* = 2.6 Hz, 1H, H_6_), 7.54 (dd, ^3^ *J* = 8.6 Hz, ^4^ *J* = 2.6 Hz, 1H, H_4_), 7.07 (d, ^3^ *J* = 8.6 Hz, 1H, H_3_), 4.59 (t, ^3^ *J* = 6.1 Hz, 2H, H_b_), 3.61 (t, ^3^ *J* = 6.1 Hz, 2H, H_c_), 2.36 (s, 3H, H_a_). ^13^C NMR (101 MHz, CDCl_3_): δ 169.5, 162.7 (2CO), 149.4 (C, C_2_), 134.1 (CH, C_4_), 132.0 (C, C_5_), 131.6 (CH, C_6_), 125.4 (CH, C_3_), 124.0 (C, C_1_), 64.7 (CH_2_, C_b_), 28.2 (CH_2_, C_c_), 21.0 (CH_3_, C_a_).

##### 3.1.2.3 Nucleophilic substitution of intermediates **5a** and **5b** with protected l‐glutamic acid (**6a** and **6b**)

Potassium carbonate (2.0–3.0 equiv.) was added to a solution of 5‐*tert*‐butoxy‐4*S*‐[(*tert*‐butoxycarbonyl)amino]‐5‐oxopentanoic acid (1.0 equiv.) in anhydrous *N,N*‐dimethylformamide (DMF, 15 mL) under nitrogen and cooled to 0 °C. After 20 min to ensure complete deprotonation, compound **5a** or compound **5b** (1.0 equiv.) was added. The mixture was allowed to warm to room temperature and stirred until complete conversion. After dilution with EtOAc, the solution was washed with saturated NaCl solution (4 × 50 mL), dried over Na_2_SO_4_, filtered, and concentrated under reduced pressure. Purification by silica gel column chromatography (pentane/CH_2_Cl_2_ 7:3, then CH_2_Cl_2_) yielded compounds **6a** and **6b**.

###### 3.1.2.3.1 5‐(2‐((2‐Acetoxybenzoyl)oxy)ethyl)‐1‐(*tert*‐butyl)(*tert*‐butoxycarbonyl)‐l‐glutamate (**6a**)

Compound **6a** (3.36 g, 6.60 mmol, 98%) was isolated as a yellow oil from compound **5a** (1.94 g, 6.75 mmol, 1.0 equiv.) and protected l‐glutamic acid (2.03 g, 6.75 mmol, 1.0 equiv.) in the presence of potassium carbonate (1.87 g, 13.5 mmol, 2.0 equiv.) after 18 h.

Rf = 0.79 (CH_2_Cl_2_). ^1^H NMR (400 MHz, CDCl_3_): δ 8.02 (d, ^3^ *J* = 7.8 Hz, 1H, H_6_), 7.55 (t, ^3^ *J* = 7.6 Hz, 1H, H_4_), 7.30 (t, ^3^ *J* = 7.6 Hz, 1H, H_5_), 7.09 (d, ^3^ *J* = 7.6 Hz, 1H, H_3_), 5.11 (d, ^3^ *J* = 8.0 Hz, 1H, NH), 4.45 (m, 2H, Hb or H_c_), 4.37 (m, 2H, H_b_ or H_c_), 4.19 (m, 1H, H_f_), 2.41 (m, 2H, H_d_), 2.33 (s, 3H, H_a_), 2.16 (m, 1H, H_e_), 1.87 (m, 1H, H_e_), 1.43, 1.41 (2 s, 18H, H_g_ and H_h_). ^13^C NMR (101 MHz, CDCl_3_): δ 172.6, 171.3, 169.6, 164.1 (4CO), 155.4 (C, C_2_), 150.8 (CO_carbamate_), 134.2 (CH, C_4_), 131.9 (CH, C_6_), 126.1 (CH, C_5_), 123.9 (CH, C_3_), 122.7 (C, C_1_), 82.1, 79.7 (2 C_*tert‐*butyl_), 62.7, 62.2 (2CH_2_, C_b_ and C_c_), 53.3 (CH, C_f_), 30.2 (CH_2_, C_d_), 28.3 (3 CH_3_, C_g_ or C_h_), 28.0 (CH_2_, C_e_), 27.9 (3 CH_3_, C_g_ or C_h_), 21.0 (CH_3_, C_a_).

###### 3.1.2.3.2 1‐(*tert*‐Butyl) 5‐(2‐((5‐chloro‐2‐hydroxybenzoyl)oxy)ethyl)(*tert*‐butoxycarbonyl)‐l‐glutamate (**6b**)

Compound **6b** (0.20 g, 0.39 mmol, 9%) was isolated (acetyl group lost) as a colorless oil by reacting compound **5b** (1.37 g, 4.26 mmol, 1.0 equiv.) and protected l‐glutamic acid (1.28 g, 4.26 mmol, 1.0 equiv.) in the presence of potassium carbonate (1.77 g, 12.8 mmol, 3.0 equiv.) after 16 h.

Rf = 0.68 (pentane/EtOAc 9:1). ^1^H NMR (400 MHz, CDCl_3_): δ 10.56 (s, 1H, OH), 7.81 (d, ^4^ *J* = 2.7 Hz, 1H, H_6_), 7.42 (dd, ^3^ *J* = 8.9 Hz, ^4^ *J* = 2.7 Hz, 1H, H_4_), 6.95 (d, ^3^ *J* = 8.9 Hz, 1H, H_3_), 5.10 (d, ^3^ *J* = 8.1 Hz, 1H, NH), 4.56 (t, ^3^ *J* = 4.8 Hz, 2H, H_a_ or H_b_), 4.47–4.41 (m, 2H, H_a_ or H_b_), 4.23 (m, 1H, H_e_), 2.53–2.36 (m, 2H, H_c_), 2.18 (m, 1H, H_d_), 1.91 (m,1H, H_d_), 1.46, 1.43 (2 s, 18H, H_f_ and H_g_). ^13^C NMR (101 MHz, CDCl_3_): δ 172.7, 171.3, 168.9 (3CO), 160.3 (C, C_2_), 155.4 (CO_carbamate_), 136.0 (CH, C_4_), 129.2 (CH, C_6_), 124.0 (C, C_5_), 119.3 (CH, C_3_), 113.0 (C, C_1_), 82.3, 79.8 (2 C_*tert*‐butyl_), 63.5, 61.9 (2CH_2_, C_a_ and C_b_), 53.2 (CH, C_e_), 30.1 (CH_2_, C_c_), 28.3 (CH_3_, C_f_ or C_g_), 28.1 (CH_2_, C_d_), 28.0 (3 CH_3_, C_f_ or C_g_).

##### 3.1.2.4 Deprotection of the α‐amino acid function of compound **6a** and **6b** to obtain conjugates **7a** and **7b**

Trifluoroacetic acid (5 mL) was added to a solution of compound **6a** (0.35 g, 0.68 mmol) or compound **6b** (0.20 g, 0.39 mmol) in anhydrous CH_2_Cl_2_ (10 mL). The reaction mixtures were stirred at room temperature for 48 h (for **6a**) or 1 h (for **6b**) until complete conversion. Evaporation under reduced pressure afforded conjugates **7a** (0.17 g, 0.55 mmol, 79%) and **7b** (0.085 g, 0.24 mmol, 63%) as white powders.

###### 3.1.2.4.1 2S‐Amino‐5‐(2‐((2‐hydroxybenzoyl)oxy)ethoxy)‐5‐oxopentanoic acid (**7a**)

Rf = 0.04 (EtOAc); mp = 73–76 °C. ^1^H NMR (500 MHz, DMSO‐*d*_6_): δ 10.54 (s, 1H, OH), 8.51 (s, 2H, NH_2_), 7.85 (dd, ^3^ *J* = 8.0 Hz, ^4^ *J* = 1.7 Hz, 1H, H_6_), 7.52 (td, ^3^ *J* = 8.4 Hz, ^4^ *J* = 1.7 Hz, 1H, H_4_), 6.98 (dd, ^3^ *J* = 8.4 Hz, ^4^ *J* = 1.0 Hz, 1H, H_3_), 6.93 (td, ^3^ *J* = 8.0 Hz, ^4^ *J* = 1.0 Hz, 1H, H_5_), 4.32 (t, ^3^ *J* = 4.8 Hz, 2H, H_a_ or H_b_), 3.90 (m, 1H, H_e_), 3.70 (t, ^3^ *J* = 4.8 Hz, 2H, H_a_ or H_b_), 2.47 (m, 1H, H_c_), 2.37 (m, 1H, H_c_), 2.00 (m, 2H, H_d_). ^13^C NMR (126 MHz, DMSO‐*d*_6_): δ 172.6, 168.8, 160.6 (3CO), 154.8 (C, C_2_), 136.1 (CH, C_4_), 130.7 (CH, C_6_), 119.8 (CH, C_5_), 117.9 (CH, C_3_), 113.7 (C, C_1_), 67.4, 59.3 (2CH_2_, C_a_ and C_b_), 51.9 (CH, C_e_), 29.6 (CH_2_, C_c_), 25.1 (CH_2_, C_d_). HRMS (ESI, methanol): *m/z* calculated for C_14_H_18_NO_7_ [M + H]^+^ 312.1083, *m/z* found 312.1071.

###### 3.1.2.4.2 2S‐Amino‐5‐[2‐[(5‐chloro‐2‐hydroxybenzoyl)oxy]ethoxy]‐5‐oxopentanoic acid (**7b**)

Rf = 0.12 (pentane/EtOAc/AcOH 5:4:2); mp = 84–87 °C. ^1^H NMR (400 MHz, DMSO‐*d*_6_): δ 7.86 (d, ^4^ *J* = 2.7 Hz, 1H, H_6_), 7.58 (dd, ^3^ *J* = 8.9 Hz, ^4^ *J* = 2.7 Hz, 1H, H_4_), 7.04 (d, ^3^ *J* = 8.9 Hz, 1H, H_3_), 4.32 (t, ^3^ *J* = 5.6 Hz, 2H, H_a_ or H_b_), 4.06 (m, 1H, H_e_), 3.73 (t, ^3^ *J* = 5.6 Hz, 2H, H_a_ or H_b_), 2.42–2.32 (m, 1H, H_d_), 2.18 (m, 2H, H_c_), 2.06–1.98 (m, 1H, H_d_). ^13^C NMR (101 MHz, DMSO‐*d*_6_): δ 177.4, 174.9, 167.8 (3CO), 156.2 (C, C_2_), 135.6 (CH, C_4_), 129.8 (CH, C_6_), 123.2 (C, C_5_) 120.0 (CH, C_3_), 114.6 (C, C_1_), 67.8, 59.2 (2CH_2_, C_a_ and C_b_), 55.2 (CH, C_e_), 29.8 (CH_2_, C_c_), 25.2 (CH_2_, C_d_). HRMS (ESI, methanol): *m/z* calculated for C_14_H_17_ClNO_7_ [M + H]^+^ 346.0688, *m/z* found 346.0695.

### 3.2 Salicylic acid–amino acid conjugates trigger defense responses in *A. thaliana*

In 1979, White reported that exogenous application of SA and acetylsalicylic acid (aspirin) induced resistance to tobacco mosaic virus in tobacco. This pioneering study provided the first evidence that SA could act as a defense‐inducing molecule, although its endogenous role in plant immunity was established later.^36^ Subsequent studies have confirmed that accumulation of SA is essential for activating defense responses against fungi, bacteria, viruses and even some insects.^37^, ^38^ A key output of SA signaling is the induction of PR proteins, among which PR1 is strongly associated with SAR.^11^, ^17^ In *A. thaliana*, *PR1* is one of the most SA‐responsive genes and is widely used as a reliable molecular marker to monitor SA‐dependent defense signaling.^39^

**Figure 4:**
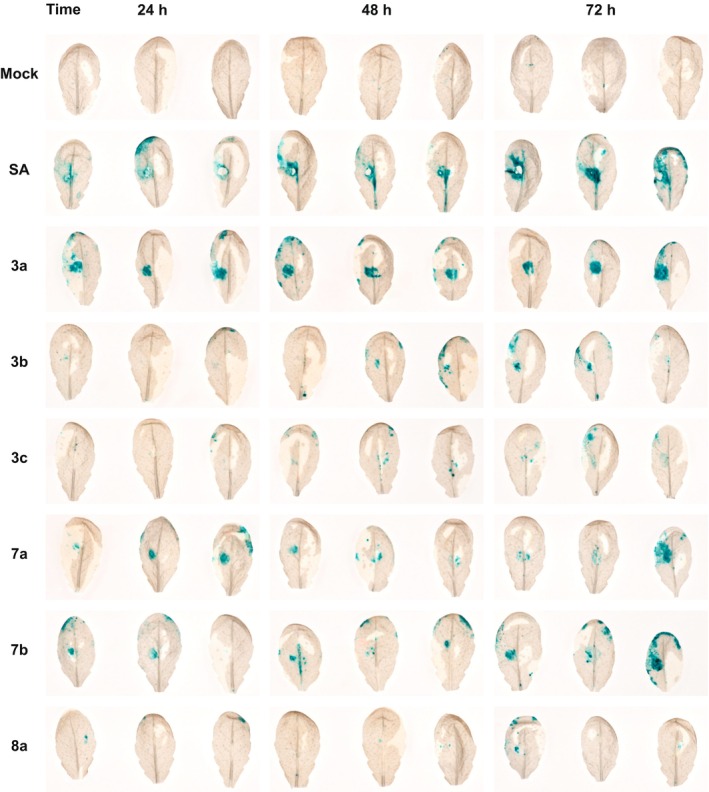
Induction of the *PR1::GUS* reporter in transgenic *Arabidopsis thaliana* following treatment with synthetic compounds **3a–3c**, **7a**, **7b**, and **8a**. Six‐week‐old *A. thaliana* plants carrying the *PR1::GUS* reporter construct (GUS gene under the control of the *PR1* promoter) were treated by applying a 10 μL droplet of each compound (1 mm) onto the adaxial surface of the left side of a leaf, at its central region. Leaves were harvested at 24, 48, and 72 h after treatment and subjected to histochemical GUS staining. Each treatment was performed in three independent biological replicates. Mock: solvent control without active compound.

**Figure 5:**
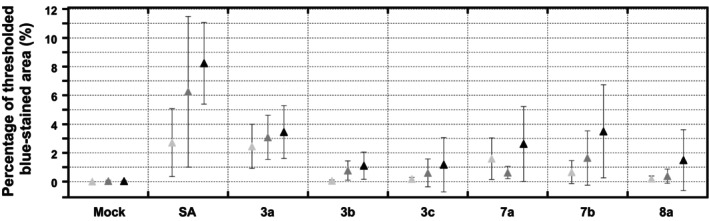
Semi‐quantitative analysis of PR1::GUS staining kinetics. The percentage of thresholded blue‐stained area was quantified at 24 (light gray), 48 (medium gray) and 72 h (black) post‐treatment using identical threshold settings for all images. Quantification was performed on six leaves per treatment and time point (two leaves from each of three independent plants) from one representative experiment. Values represent mean ± standard deviation.

To evaluate whether the newly synthesized SA–amino acid conjugates can activate SA‐dependent defenses, transgenic *A. thaliana PR1::GUS* reporter plants were used. In this system, the GUS reporter gene is driven by the *PR1* promoter, allowing sensitive and spatially resolved visualization of *PR1* gene expression through histochemical staining. Each compound was applied as a 10 μL droplet to the adaxial surface of a leaf, and GUS staining was monitored at 24, 48, and 72 h post‐treatment (Fig. 4). As expected, SA triggered strong GUS staining both at the application site and along the veins, indicating activation of *PR1* not only locally but also in distal leaf tissues. This vascular‐associated staining is consistent with SA mobility and its ability to induce systemic signaling. The intensity of GUS staining increased progressively from 24 to 72 h, consistent with a sustained activation of PR1 over time, as supported by semi‐quantitative image analysis (Fig. 5).

All SA–amino acid conjugates tested induced *PR1* expression with distinct intensities and expression patterns. This behavior parallels that of salicyloyl–aspartate, the only endogenous SA–amino acid conjugate known in plants, which similarly triggers *PR1* induction in *A. thaliana*.^40^ Notably, each conjugate generated a specific *PR1* induction profile, both in magnitude and temporal dynamics (Figs 4 and 5). Among the diaminocarboxylic acid series, conjugate **3a** elicited the strongest induction, evidenced as intense GUS staining at the application site as early as 24 h post‐treatment. Semi‐quantitative analysis confirmed its early and sustained induction profile, with consistently higher stained areas compared to the other conjugates (Fig. 5). However, in contrast to free SA, conjugate **3a** did not induce any detectable distal vein‐associated staining, suggesting limited mobility or slower release of SA from the conjugate. Conjugates **3b** and **3c** also activated *PR1* expression, but their staining was weaker and appeared later, mostly between 48 and 72 h (Fig. 5). This progressively delayed and attenuated response correlates with the increasing length of the amino acid carbon chain (Fig. 3), in line with previous observations indicating that shorter linkers favor mobility or availability of active SA derivatives.^30^

Within the l‐glutamic acid conjugates series, compounds **7a** and **7b**, both carrying an ethylene glycol spacer arm, induced PR1 expression with a more progressive kinetic profile. While early induction at 24 h remained moderate, staining intensity increased at later time points, and by 72 h their activity approached that observed for conjugate **3a** (Fig. 5). However, this late response was associated with higher variability, suggesting less homogeneous or less synchronized activation across leaves. The presence or absence of a chlorine atom on the SA moiety did not markedly affect their overall induction pattern. In contrast, conjugate **8a**, which contains a 1,2,3‐triazole spacer, produced little to no detectable GUS staining in *A. thaliana*, despite having previously been shown to induce *ZmPR1* expression in maize.^28^ This low activity was confirmed by the limited stained area measured at all time points (Fig. 5). These contrasting outcomes highlight that the biological activity of SA conjugates can differ markedly between plant species, likely reflecting variations in uptake, transport, metabolic processing or enzymatic release of free SA.

Taken together, these results show that while all conjugates retain some ability to activate SA‐dependent defenses, their efficacy differs markedly according to their structural features. Conjugate **3a**, carrying the shortest side chain and an amide linkage, emerged as the most potent inducer of *PR1* expression. Conjugates **7a** and **7b**, linked through ester bonds, elicited moderate and localized responses, whereas conjugate **8a** was largely inactive in *A. thaliana*. Overall, these findings indicate that both the chemical nature of the linkage (amide *versus* ester *versus* triazole) and the physicochemical properties of the amino acid moiety play decisive roles in shaping the intensity and pattern of defense activation *in planta*.

### 3.3 Salicylic acid–amino acid conjugates reduce phytotoxicity and protect *A. thaliana* against *P. syringae*

**Figure 6:**
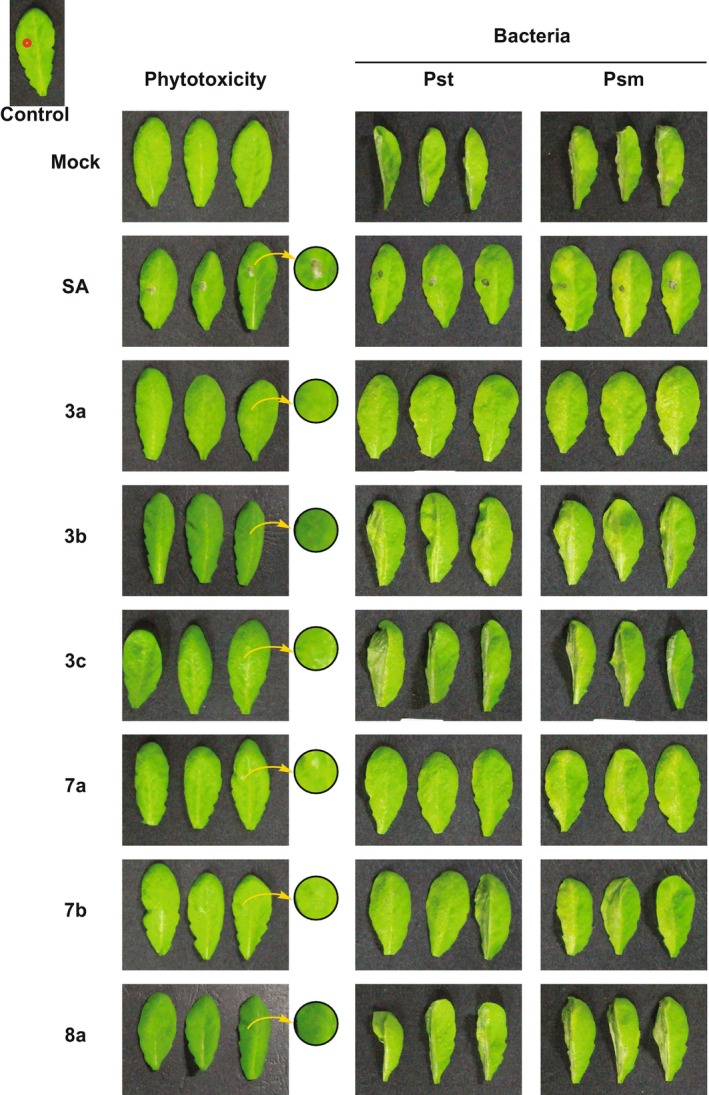
Phytotoxicity and bacterial disease response in *Arabidopsis thaliana* following treatment with synthetic compounds **3a–3c**, **7a**, **7b**, and **8a**. Six‐week‐old *A. thaliana* Col‐0 plants were treated with a 10 μL droplet of each compound at 1 mm concentration, applied to the center of the left side of a leaf on the adaxial (upper) surface. Phytotoxicity symptoms were documented 72 h post‐treatment. For disease assessment, leaves were infiltrated with a bacterial suspension (10^8^ CFU mL^−1^) 2 days after treatment, and symptoms were photographed 48 h post‐infection. Insets show a magnified view of the droplet application site, allowing direct comparison of SA‐induced localized necrosis with the absence of visible phytotoxicity for the SA–amino acid conjugates. The experiment was repeated three times with consistent results. Control: untreated and uninfected (the site of droplet application is indicated by a red circle); Mock: dilution solution without compound; Pst: infected with *Pseudomonas syringae* pv. *tomato* DC3000; Psm: infected with *Pseudomonas syringae* pv. *maculicola*.

Before evaluating their protective effects against *P. syringae*, we first examined whether the synthesized conjugates induced visible phytotoxicity when applied on *A. thaliana* leaves. Phytotoxicity is a major limitation of exogenous SA application, which frequently causes necrotic lesions at deposition sites, even at moderate concentrations.^22^, ^24^ Mature Col‐0 leaves were treated with a 10 μL droplet of each compound at 1 mm, and symptoms were recorded 3 days later (Fig. 6, first column). To facilitate visual assessment of localized damage at the site of compound application, a magnified inset (‘zoom’) of the droplet deposition area is shown for each treatment in Fig. 6. These insets enable direct comparison of local tissue responses independently of infection symptoms. These insets clearly reveal that exogenous SA induced marked localized necrosis at the application site, consistent with previous reports showing that millimolar SA can cause chlorosis, growth inhibition, or tissue necrosis across diverse plant species, including both monocots and dicots.^22^, ^23^, ^24^, ^25^ These phytotoxic effects are commonly attributed to SA‐mediated disruptions in cellular redox homeostasis and the resulting overaccumulation of reactive oxygen species. In striking contrast, none of the SA–amino acid conjugates evaluated in this study (compounds **3a–3c**, **7a**, **7b** and **8a**) caused any visible phytotoxic effect, irrespective of the amino acid used, the linker type, or the position of attachment on the SA scaffold. These observations clearly indicate that vectorizing SA through amino acid conjugation markedly reduces or abolished SA‐induced phytotoxicity, supporting the rationale of the prodrug approach. This absence of toxicity is consistent with earlier observations of Chen *et al*., who demonstrated that salicyloyl–aspartate caused substantially fewer lesion damage than free SA at 1 mm, and that the damage caused by 1 mm SA was comparable to that caused by 3 mm salicyloyl–aspartate.^40^

To determine whether *PR1* induction by the synthesized conjugates translates into effective disease resistance, we evaluated their protective activity against the virulent foliar pathogens Pst, the causal agent of bacterial speck, or Psm, responsible for bacterial blight/leaf spot on crucifers (Fig. 6, columns 2 and 3). Untreated and non‐infiltrated leaves were used as controls. Mock‐treated leaves, infiltrated with a high inoculum in the absence of active compounds, developed severe disease symptoms, including rapid wilting, leaf softening, tissue collapse, intense chlorosis, water‐soaked lesions, localized necrosis, and accelerated senescence, affecting the infiltrated half of the leaf (Fig. 6, Bacteria). These responses were consistently observed for both pathogens, confirming their virulence under our experimental conditions. As expected, and despite a slightly more pronounced chlorosis following inoculation with Psm compared with Pst, SA markedly reduced the development of typical disease symptoms in Col‐0 leaves, although phytotoxic lesions remained similar to those described earlier.

In comparison, leaves treated with conjugates **3a** and **7a** exhibited a strong protective effect, with disease symptoms remaining minimal and closely comparable to those observed following treatment with free SA. Importantly, these conjugates did not induce the localized phytotoxic lesions associated with SA, indicating a markedly improved toxicity profile at this concentration. Treatment with conjugates **3b** and **7b** resulted in a moderate protection: disease development was reduced relative to the mock control, but remained more pronounced than in SA‐, **3a‐** or **7a**‐treated leaves. As with the most active conjugates, no phytotoxicity was observed for compound **3b** or compound **7b**. In contrast, conjugates **3c** and **8a** failed to confer any detectable protection, as symptoms on treated leaves were similar to those of mock‐treated plants.

**Figure 7:**
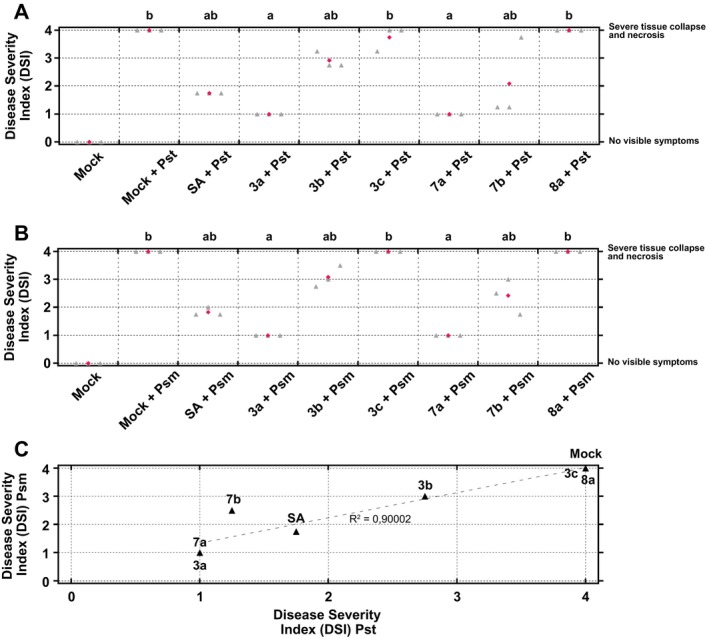
Quantitative assessment of disease severity and concordance between Pst and Psm responses. (A, B) DSI in *Arabidopsis thaliana* Col‐0 leaves treated with SA and synthetic compounds **3a**–3**c**, **7a**, **7b**, and **8a** (1 mm), then subsequently infected with Pst (A) or Psm (B), as described in Fig. 6. Disease symptoms were scored 48 h post‐infection using a 0–4 DSI, where 0 = no visible symptoms (healthy, green, turgid tissue), 1 = very mild diffuse chlorosis without necrosis, 2 = moderate chlorosis with beginning loss of turgor but largely intact tissue, 3 = severe chlorosis with tissue softening and initial localized necrosis, and 4 = extensive necrosis and tissue collapse comparable to infected mock controls. Each point represents the mean DSI of a single leaf scored independently by four observers (*n* = three leaves per treatment) and red symbols indicate the median DSI per treatment; inter‐observer agreement for DSI scoring was assessed using Kendall's coefficient of concordance (*W*), which indicated strong agreement among scorers. Different letters above the plots denote statistically significant differences (Kruskal–Wallis test followed by Dunn's *post hoc* test with FDR‐BH correction, *α* = 0.05). (C) Concordance of DSI between Pst and Psm infections. Scatter plot of median DSI values for each treatment in Pst (*x*‐axis) *versus* Psm (*y*‐axis). Each point corresponds to one treatment. A linear regression line is shown for visualization. Concordance between the two pathogens was assessed using Spearman's rank correlation (*ρ =* 0.975, *P* < 0.01), indicating a strong positive relationship between disease severity induced by Pst and Psm across treatments.

To complement this qualitative assessment, disease severity was quantified using a standardized DSI scale (0–4), based on visual scoring of chlorosis, tissue integrity, and necrosis. This quantitative approach (Fig. 7(A),(B)) corroborated the visual patterns observed in Fig. 6: treatments of compounds **3a** and **7a** consistently exhibited the lowest DSI values (highest protection), whereas compounds **3c** and **8a** clustered with the infected mock control (highest DSI). Intermediate DSI values were obtained for SA, compound **3b**, and compound **7b**, confirming their partial protective effect. Statistical analysis of the DSI scores confirmed significant differences among treatments for both Pst and Psm infections, with compounds **3a** and **7a** forming a distinct low‐DSI group compared to the infected mock and inactive conjugates. Inter‐observer agreement for DSI scoring was high, supporting the robustness of the visual assessment.

These phenotypic observations were fully consistent with the histochemical GUS assay results assessing *PR1* promoter activity. Enhanced *PR1* expression correlated strongly with improved protection against bacterial infection. Specifically, conjugates **3a** and **7a** induced robust *PR1* activation, in agreement with their protective effects, whereas compounds **3b** and **7b** triggered moderate *PR1* expression, and compounds **3c** and **8a** remained largely inactive. Taken together, these results highlight a clear structure–activity relationship, in which the amino acid moiety and the nature of the spacer arm critically determine the ability of the conjugates to modulate SA‐responsive defense pathways such as *PR1* induction. It is noteworthy that only l‐configured amino acids were used in the present study. This choice was guided by our previous work^29^, ^41^ demonstrating a clear stereospecific recognition of amino acid–xenobiotic conjugates by plant transport systems, where l‐conjugates showed efficient uptake and systemic transport, whereas the corresponding d‐forms exhibited markedly reduced activity. These findings support the relevance of focusing on l‐configured conjugates when designing transporter‐targeted prodrugs in plants. The ability of certain SA conjugates to activate plant defenses has been reported previously. For example, SA–pyroglutamic acid conjugates were shown to protect bread wheat against the phytopathogenic fungus *Zymoseptoria tritici*,^42^ and salicyloyl–aspartate was reported to induce *PR1* expression and enhance resistance to Pst.^40^ The behavior of conjugates **3a** and **7a** is consistent with these findings, reinforcing the notion that amino acid vectorization can preserve – or even enhance – SA‐dependent defense activation.

By contrast, conjugate **8a**, which previously showed significant protective effect in maize – both locally and systemically against the pathogenic fungi *B. maydis* and *F. graminearum* – and induced *ZmNPR1* and *ZmPR1* expression upon challenge with *B. maydis*, exhibited almost no *PR1* induction and no protective activity in *A. thaliana*. These contrasting results highlight that the efficacy of SA conjugates is highly dependent on plant species, likely reflecting differences in uptake, transport or metabolism in *Arabidopsis*, or alternatively, an inability of the plant to efficiently cleave the conjugate to release free SA.

To further compare pathogen‐specific efficacy, we examined the relationship between DSI values obtained for Pst and Psm across all treatments (Fig. 7(C)). A strong positive correlation was observed, indicating that compounds conferring protection against one *P. syringae* pathovar tended to show comparable effectiveness against the other. This concordance supports the robustness of the observed structure–activity relationships and suggests that the mode of action of the most active conjugates (**3a** and **7a**) is largely conserved between these two pathogens.

Overall, the superior performance of conjugates **3a** and **7a** – characterized by strong protection, robust *PR1* activation, and absence of phytotoxicity – demonstrates the potential of amino acid vectorization to deliver SA‐mediated immunity while avoiding the detrimental side effects associated with exogenous SA application.

### 3.4 The protective effect did not result from direct antibacterial activity

**Figure 8:**
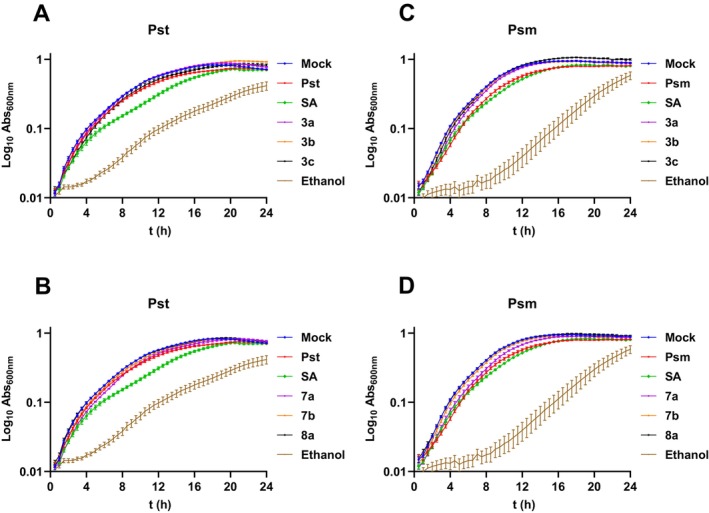
*In vitro* growth of Pst (A, B) and Psm (C, D) in the presence of synthetic compounds **3a–3c**, **7a**, **7b** and **8a**. Ethanol (70%) was included as a positive control, as it partially delays bacterial growth under these experimental conditions. Bacteria were grown in LB medium supplemented with compounds at 1 mm. Absorbance at 600 nm was measured every 30 min for 24 h at 28 °C. Each condition included six technical replicates; bacterial growth is expressed as log_10_(absorbance at 600 nm); data shown represent one of four independent biological replicates. Mock: dilution solution without compound; Pst: *Pseudomonas syringae* pv. *tomato* DC3000; Psm: *Pseudomonas syringae* pv. *maculicola*.

To determine whether the protective effects observed *in planta* resulted from the activation of plant immune responses – such as *PR1* induction – or from a direct antimicrobial activity of SA or its synthesized conjugates, we evaluated the impact of these compounds on the *in vitro* growth of the two bacterial pathogens used in this study. As shown in Fig. 8, at 1 mm, neither SA nor any of the conjugates significantly inhibited the growth of Pst or Psm. The growth kinetics of both strains, including the lag, exponential and stationary phases, were virtually identical to those of the mock controls, indicating that these compounds did not affect any stage of bacterial multiplication under the tested conditions. In contrast, the ethanol control, included as a positive growth inhibitor, induced a pronounced lag phase, confirming the sensitivity and robustness of the assay. These findings are consistent with previous work. Guichard *et al*. demonstrated that at 1 mm, neither SA nor conjugate **8a** significantly inhibited the mycelial growth of *B. maydis*, supporting the view that these compounds do not exhibit direct fungicidal activity at this concentration.^28^ Similarly, Mejri *et al*. reported that SA–pyroglutamic acid conjugates protected wheat against *Zymoseptoria tritici* despite showing no detectable direct antifungal activity; in their study, SA only inhibited fungal growth at much higher concentrations, with an estimated half‐maximal inhibitory concentration (IC_50_) of approximately 3.2 mm and clear growth inhibition occurring at 5 mm.^42^

Other studies investigating the antimicrobial properties of SA have reported minimum inhibitory concentrations (MICs) well above the 1 mm used here. For example, Karpiński and Ożarowski reported MIC values of 0.31–1.25 mg mL^−1^ (2.24–9.05 mm) against several foodborne bacterial pathogens, including *Staphylococcus aureus*, *Listeria monocytogenes*, *Escherichia coli*, and *Salmonella enterica* serovar Typhimurium.^43^ Adamczak *et al*. similarly reported MIC values of 250–500 μg mL^−1^ (1.81–3.62 mM) for inhibitory effects of SA on *Escherichia coli*, *Pseudomonas aeruginosa*, *Enterococcus faecalis*, and *S. aureus*.^44^

Taken together, these results demonstrate that the 1 mm concentration used in our biological assays is below the threshold required to exert direct antimicrobial effects on the two *P. syringae* strains tested. This absence of inhibitory activity was consistent across all synthesized conjugates. Thus, the protective effects observed *in planta* for SA and the most active conjugates (**3a** and **7a**) are most likely mediated by the stimulation of SA‐dependent plant immune responses, including *PR1* induction, rather than by direct antimicrobial effects.

### 3.5 NPR1 was required for the protective effect of SA conjugates

NPR1 plays a central role in SA‐mediated signaling,^17^, ^45^ and is recognized as an SA receptor whose activation by SA is required for the transcriptional induction of SA‐responsive genes such as *PR1*.^46^, ^47^ This regulatory role is conserved across plant species, including rice.^48^

In this study, SA and several of its amino acid conjugates induced *PR1* expression and conferred protection against *P. syringae* in *A. thaliana* wild‐type plants. To determine whether these effects depend on NPR1, we analyzed *npr1* mutant leaves treated with SA or the conjugates prior to bacterial infection. Consistent with previous reports, *PR1* expression requires both SA accumulation and NPR1 function, and the *npr1* mutant fails to express PR1 following SA treatment.^32^ This mutation disrupts the expression of PR genes and abolishes SAR.^17^, ^32^, ^49^

**Figure 9:**
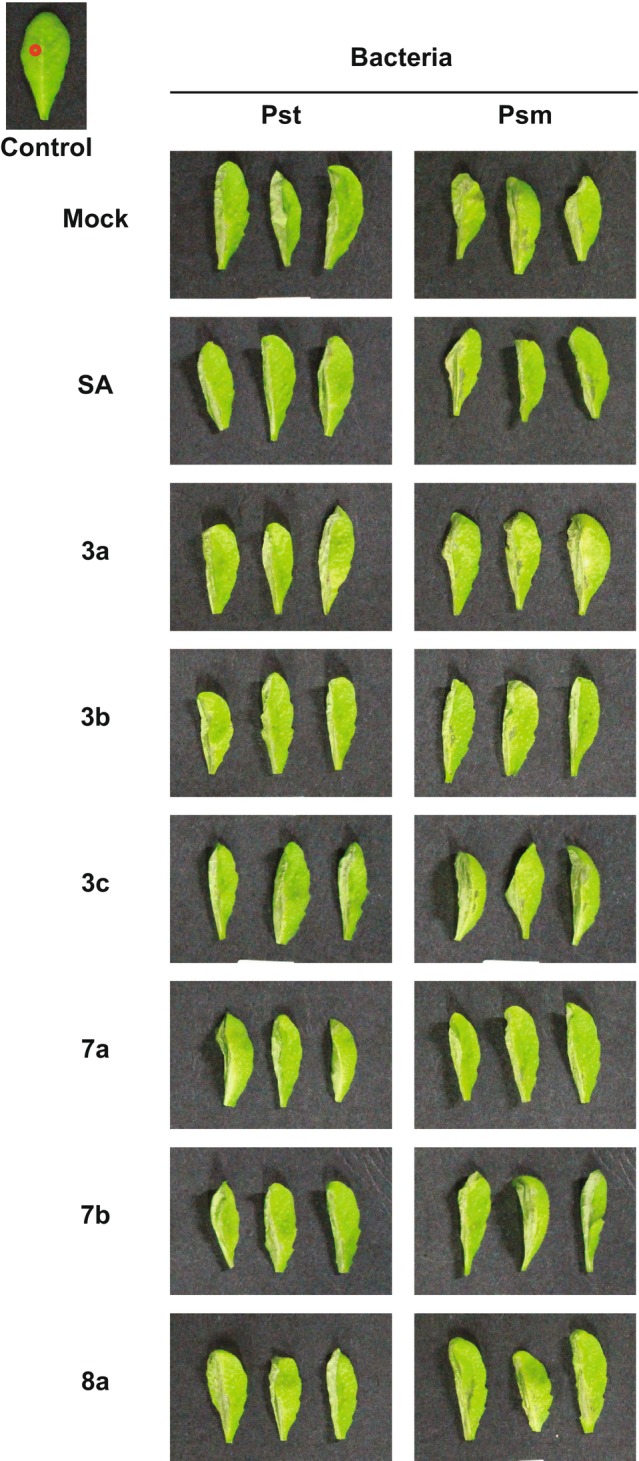
Bacterial disease response in *Arabidopsis thaliana npr1* mutant following treatment with synthetic compounds **3a–3c**, **7a**, **7b**, and **8a**. Six‐week‐old *A. thaliana* NPR1‐deficient mutant plants were treated with a 10 μL droplet of each compound at 1 mm concentration, applied to the center of the left side of a leaf on the adaxial (upper) surface. Two days later, leaves were infiltrated with a bacterial suspension (10^8^ CFU mL^−1^), and disease symptoms were observed 48 h post‐infection. The experiment was repeated three times with consistent results. Control: untreated and uninfected (the site of droplet application is indicated by a red circle); Mock: dilution solution without compound; Pst: infected with *Pseudomonas syringae* pv. *tomato* DC3000; Psm: infected with *Pseudomonas syringae* pv. *maculicola*.

Accordingly, *npr1* mutant leaves treated with SA or with any of the conjugates displayed extensive disease symptoms after inoculation, including wilting and tissue collapse of the infiltrated half‐leaf (Fig. 9). These symptoms were indistinguishable from those observed in mock‐treated mutant leaves. In contrast to wild‐type plants, neither SA nor the amino acid conjugates conferred measurable resistance in the absence of functional NPR1.

These results confirm that the protective activity of SA–amino acid conjugates relies on NPR1, just as SA‐mediated defenses do. Similar NPR1 dependence has been reported for other functional SA analogs. For example, 71% of INA (2,6‐dichloroisonicotinic acid)‐induced genes are impaired in the *npr1* mutant,^49^, ^50^ and the defense activator probenazole (PBZ) also acts through the SA/NPR1 signaling pathway, inducing NPR1 accumulation and activating SA‐dependent genes.^51^, ^52^

Altogether, these findings indicate that NPR1 constitutes a central signaling hub required not only for SA and classical analogs, but also for the biological activity of SA–amino acid conjugates. Their protective effect thus arises from their ability to contribute to SA/NPR1‐dependent immune signaling rather than from an NPR1‐independent mechanism.

## 4 CONCLUSION

In this study, six SA–amino acid conjugates were synthesized and evaluated for their ability to activate SA‐dependent defense responses and protect *A. thaliana* against *P. syringae* infection. Three conjugates were generated by linking SA to diaminocarboxylic acids of different chain lengths without a spacer arm. Two additional conjugates were obtained by coupling SA or a mono‐halogenated SA analog to l‐glutamic acid through an ethylene glycol spacer and a sixth conjugate, previously reported, incorporated a 1,2,3‐triazole ring between SA and l‐glutamic acid. All compounds were successfully synthesized with good overall yields. Biological assays revealed a clear structure–activity relationship. Conjugates **3a** and **7a** showed the strongest activity, inducing *PR1* expression and conferring protection comparable to free SA against both Pst and Psm. Conjugates **3b** and **7b** displayed moderate and/or delayed *PR1* induction associated with weaker or more variable protection. Conjugate **3c** induced only marginal *PR1* expression and lacked protective activity. Interestingly, conjugate **8a**, previously shown to be active in maize, remained inactive in *A. thaliana*, highlighting potential species‐dependent differences in uptake, transport, or metabolic processing of SA conjugates. Importantly, none of the synthesized conjugates caused visible phytotoxicity at 1 mm, in contrast to free SA, which consistently induced localized tissue necrosis. For the most active conjugates **3a** and **7a**, the protective effect was abolished in the *npr1* mutant, demonstrating that their activity relies on SA/NPR1‐mediated signaling.

Together, these results highlight that both the nature of the bond linking SA to the amino acid and the structural features of the amino acid side chain strongly influence biological activity. The high efficacy of conjugates **3a** and **7a**, combined with their absence of phytotoxicity, underscores the potential of amino acid‐based vectorization strategies for developing SA prodrugs that enhance plant immunity without causing tissue damage.

Further investigation will be essential to elucidate the mechanisms underlying the release, transport, and perception of these conjugates within plant cells. Expanding this chemical space through the rationale design and synthesis of additional SA–amino acid conjugates will help build a comprehensive library of candidates, supporting the identification of optimized SA‐based defense inducers suitable for integration into sustainable crop protection strategies.

## Data Availability

The data that support the findings of this study are available from the corresponding author upon reasonable request.
